# Supplementary material for: Mechanical properties of atomically thin boron nitride and the role of interlayer interactions
Source: Nat Commun. 2017 Jun 22;8:15815. doi: 10.1038/ncomms15815 (PMC5489686; doi:10.1038/ncomms15815)
Supplement: Supplementary Information — Supplementary Figures and Supplementary References [file ncomms15815-s1.pdf]

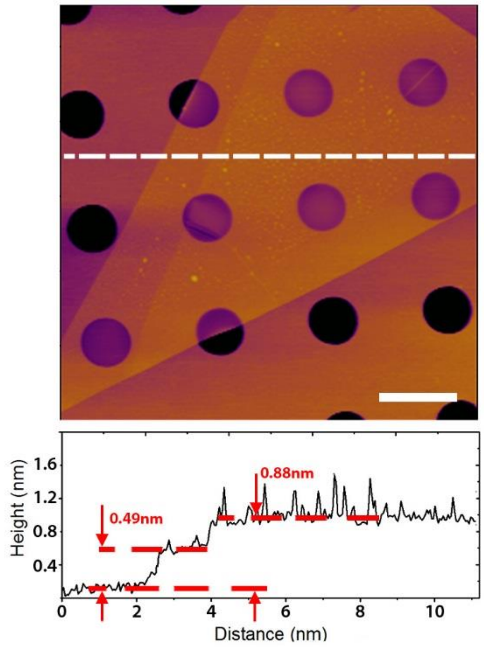

Supplementary Figure 1. Few-layer BN nanosheets. AFM image and the corresponding height trace of a 2L BN mechanically exfoliated on SiO_2/Si with pre-fabricated micro-wells. Scale bar 2 µm.

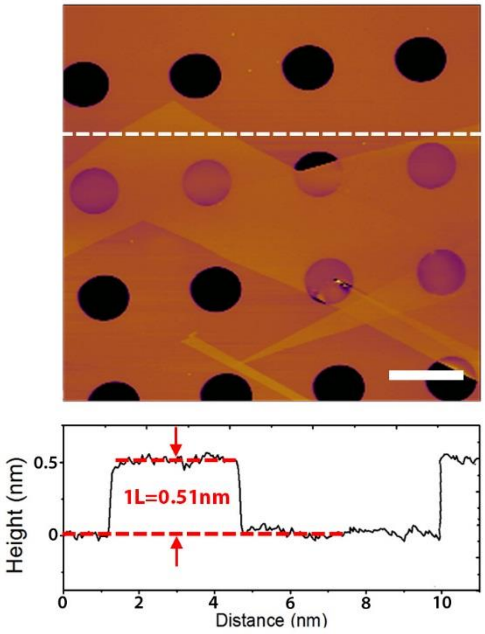

Supplementary Figure 2. Images of 1L and few-layer graphene. AFM image and the corresponding height trace of a 1L graphene on SiO_2/Si substrate with pre-fabricated micro-wells. The graphene samples were mechanically exfoliated from highly oriented pyrolytic graphite (HOPG) onto the substrate. Scale bar 2 µm.

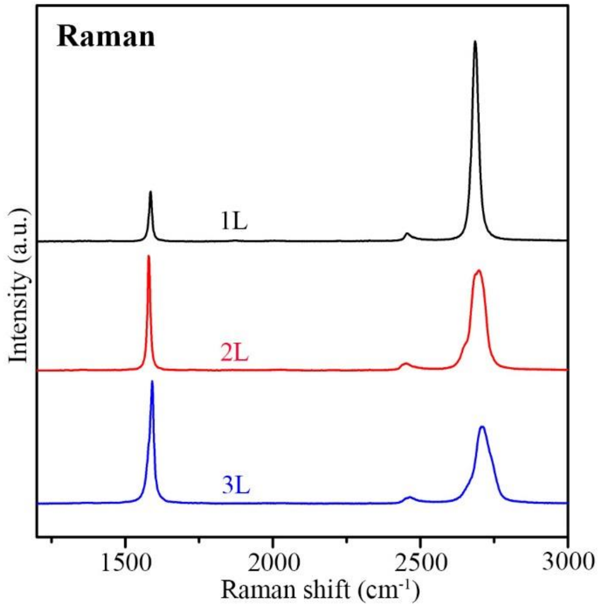

Supplementary Figure 3. Raman of 1-3L graphene. Raman spectra of the mechanically exfoliated graphene 1-3L graphene. For the Raman measurements, the laser power was 514.5 nm, and a 100x objective lens was used. The spectra confirm their thickness, and the absence of D band testifies their high quality.

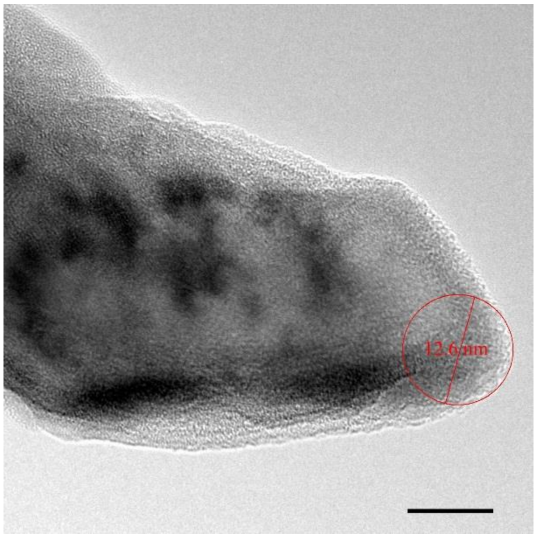

Supplementary Figure 4. Diamond tip. The diameter of the diamond tips was measured by transmission electron microscopy (TEM). This TEM image shows the tip diameter to be 12.6 nm. Scale bar 10 nm.

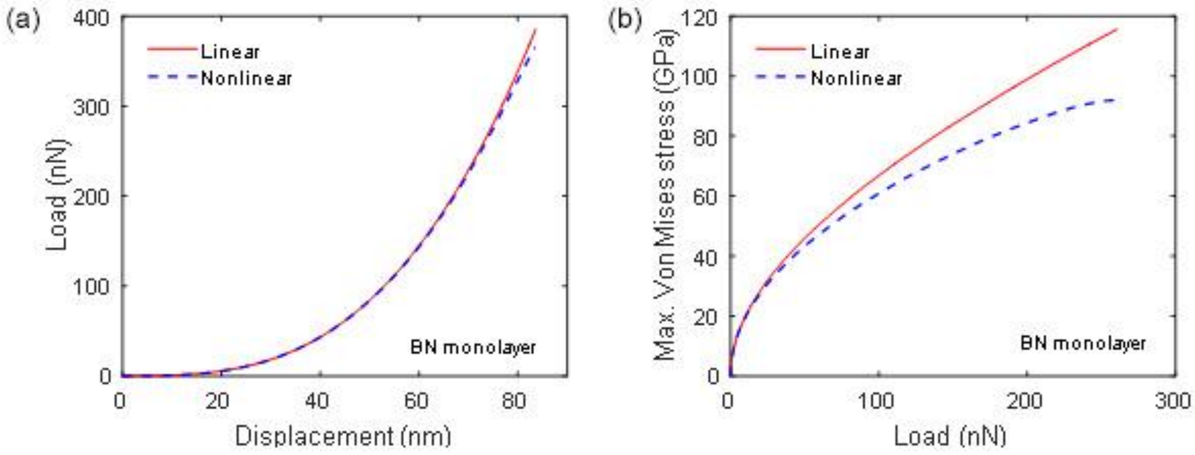

Supplementary Figure 5. Linear and nonlinear elastic constitutive behavior of 1L BN. Comparison of linear and nonlinear elastic constitutive model (a) load *vs* displacement curves and (b) maximum Von Mises stress *vs* load curves. The load-displacement responses of the linear and nonlinear elastic models can be seen in (a) to agree well with each other, except for a slight difference at the end of the loading, when the portion of nanosheets under the indenter is highly strained. On the other hand, the stress responses shown in (b) differ significantly between the two cases: the linear elastic model overestimates the stress value about 25.7% at the maximum load and hence overestimated strength values.

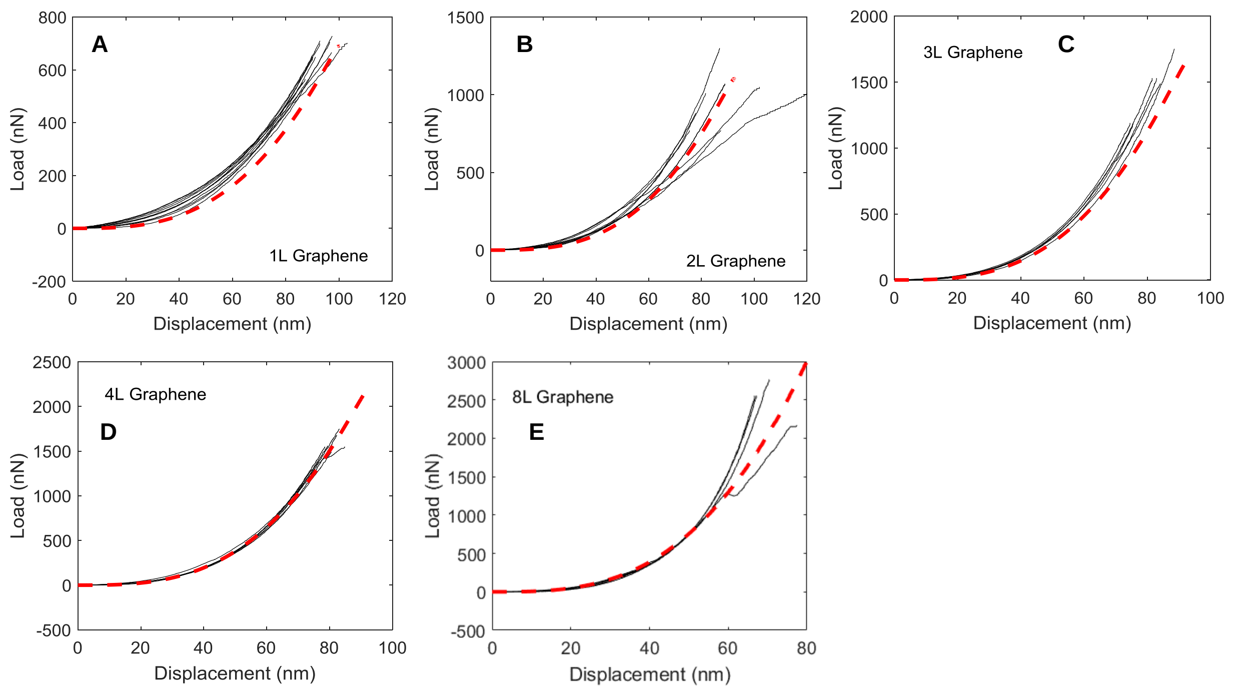

Supplementary Figure 6. Load-displacement curves for graphene nanosheets of different thicknesses. Solid and dashed lines are experimental and numerical results, respectively. The numerical predictions from the finite element model generally agree well with experimental results for all layer numbers.

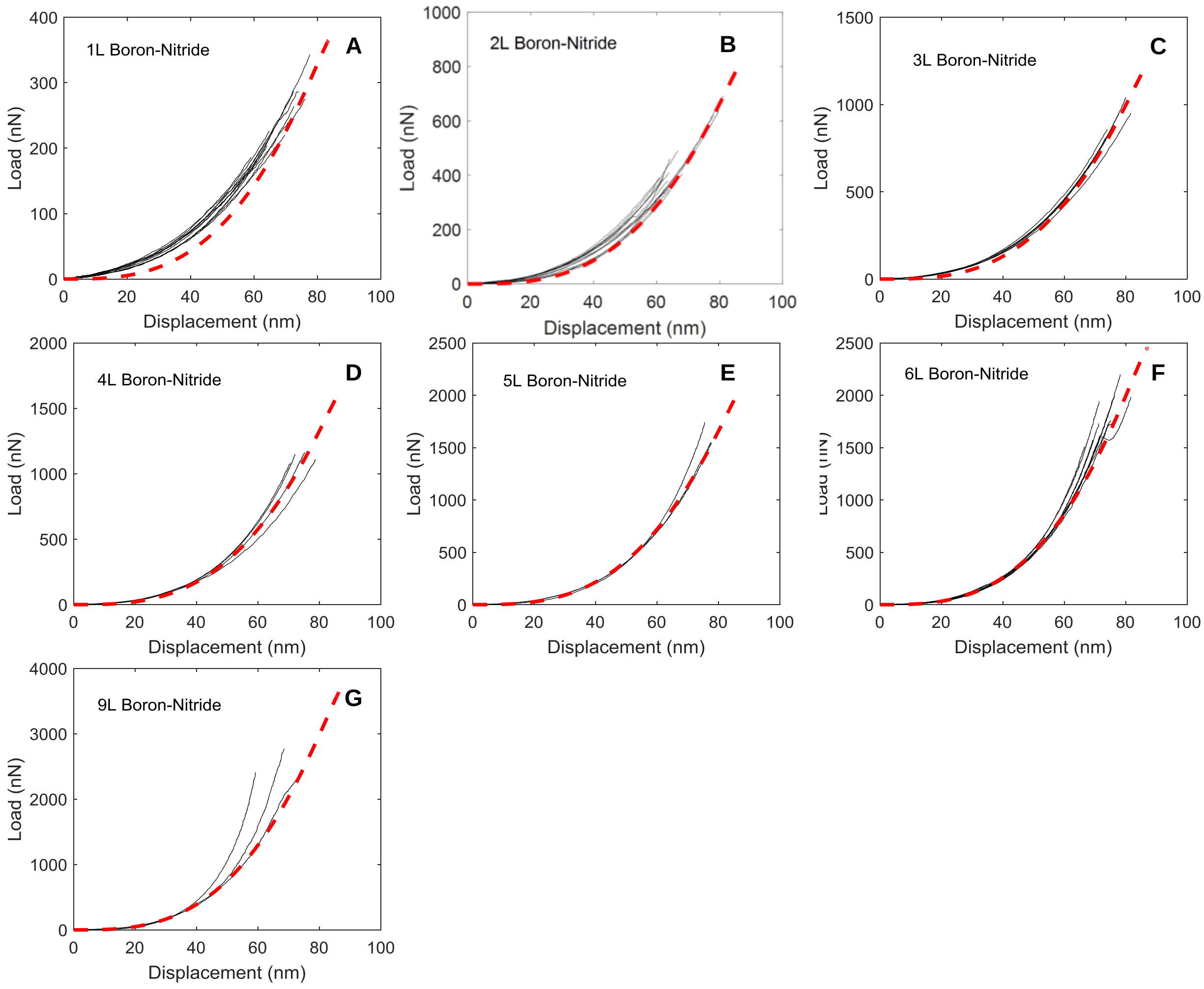

Supplementary Figure 7. Load-displacement curves for boron nitride nanosheets of different thicknesses. Solid and dashed lines are experimental and numerical results, respectively. The numerical predictions from the finite element model generally agree well with experimental results for all layer numbers.

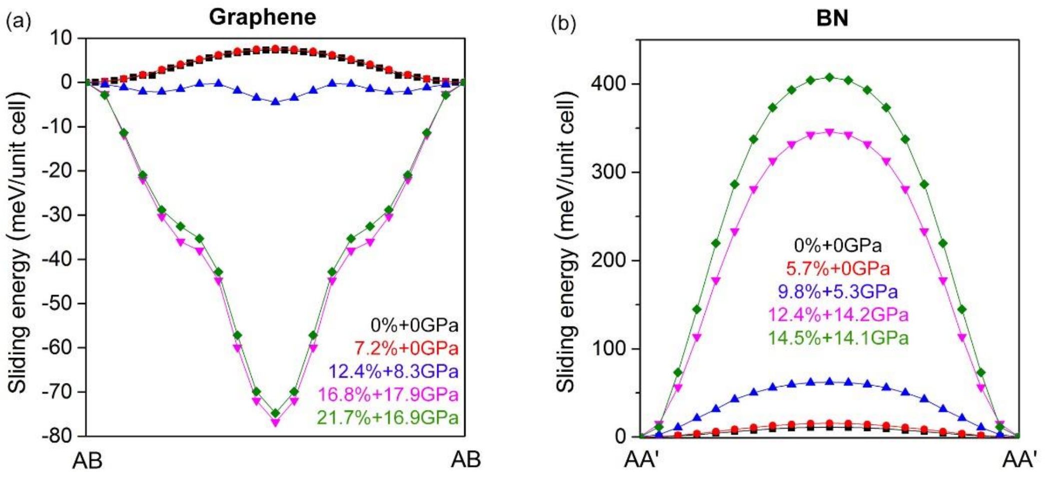

Supplementary Figure 8. Sliding energy in graphene and BN calculated by PBE+MBD. Besides the simulation using optB88-vdW functional, we also used PBE+MBD[1] to calculate the change of the sliding energy in 2L graphene and BN under the same sets of strain and pressure conditions. The PBE+MBD calculations reproduced the basic result from optB88-vdW functional that the sliding energy in graphene decreased dramatically while that in BN kept increasing with increased in-plane strain and out-of-plane compression. Sliding energy (PBE+MBD) in 2L (a) graphene under different combinations of in-plane strain (%) and out-of-plane pressure (GPa) (i.e. 0%+0GPa, 7.2%+0GPa, 12.4%+8.3GPa, 16.8%+17.9GPa, and 21.7%+16.9GPa) and (b) BN under different combinations of in-plane strain (%) and out-of-plane pressure (GPa) (i.e. 0%+0GPa, 5.7%+0GPa, 9.8%+5.3GPa, 12.4%+14.2GPa, and 14.5%+14.1GPa).

SUPPLEMENTARY NOTE 1:

The effect of pure in-plane strain or out-of-plane pressure on the sliding energy in graphene and BN

To elucidate the different effects from in-plane strain and out-of-plane pressure on the sliding energy in graphene and BN, we did PBE+MBD[1] calculations under pure strain or pure pressure conditions.

In the case of graphene, we show that when no strain is applied onto the system, the pressure (compression) has a key factor in changing the energy profile from barriers near ~ +7 meV per unit cell at 0 GPa, to values in the range of ~ -1.5 to -6.0 meV per unit cell at 17.9 GPa (Supplementary Figure 9a). Several meta-stable energy positions are also observed along the AB-AB path, indicating the strong interactions between p_z-orbitals at different points of the sliding path. We have also performed simulations under pure strain without compression, which show a slight increment of the sliding energy barrier (Supplementary Figure 10a). When both strain and pressure are included (Supplementary Figure 8a), the sliding energy in graphene further decreased dramatically. These results strongly illustrate that both strain and pressure play together in the change of the sliding energy in graphene: pressure (compression) changes the sign of the energy barrier, while strain increases the magnitude in negative energy (i.e. further downhill).

In the case of BN, both pure out-of-plane pressure and pure in-plane strain give rise to systematic increases of sliding energy (Supplementary Figure 9b and 10b), but the combination of pressure and strain further increases such barrier. In other words, both strain and pressure play together to enhance the sliding energy barriers (Supplementary Figure 8b).

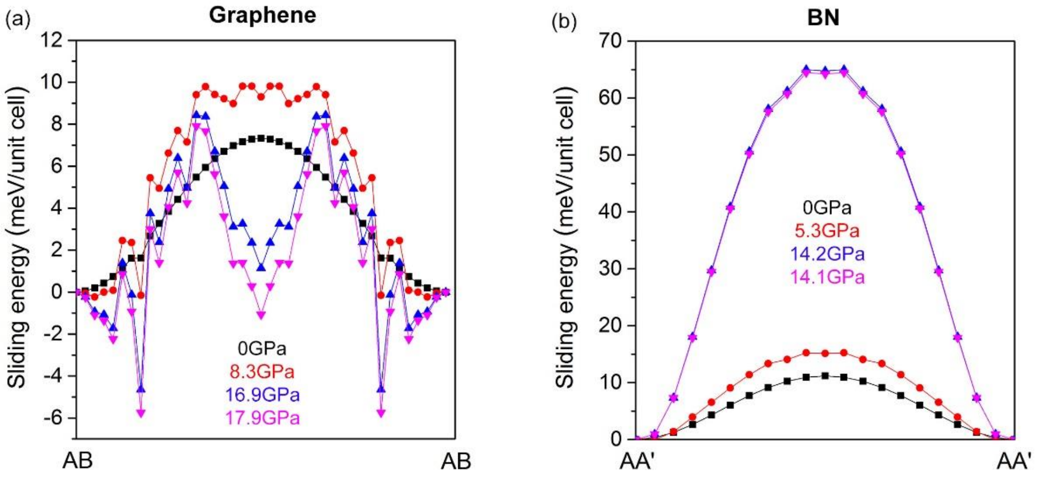

Supplementary Figure 9. Effect of pressure. The effect of pure out-of-plane pressure (without in-plane strain) on the sliding energy in 2L (a) graphene and (b) BN calculated using PBE+MBD approach.

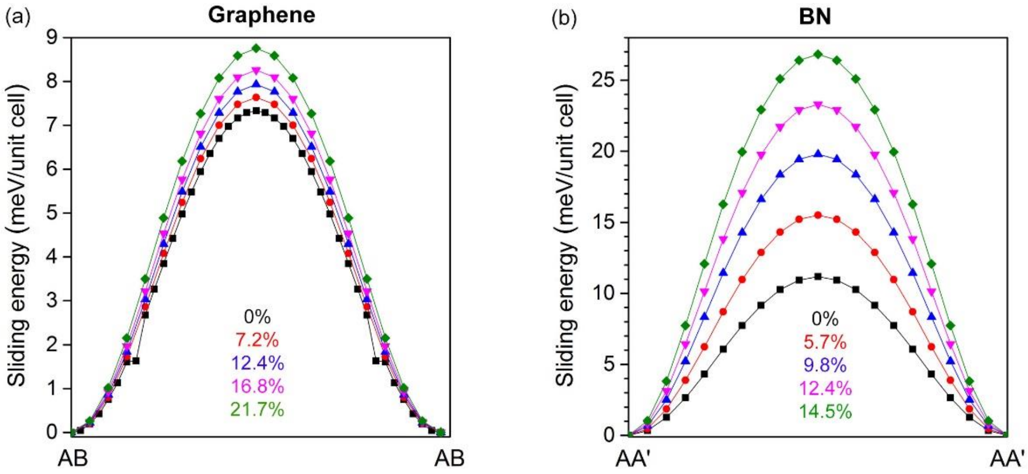

Supplementary Figure 10. Effect of strain. The effect of pure in-plane strain (without out-of-plane pressure) on the sliding energy in 2L (a) graphene and (b) BN.

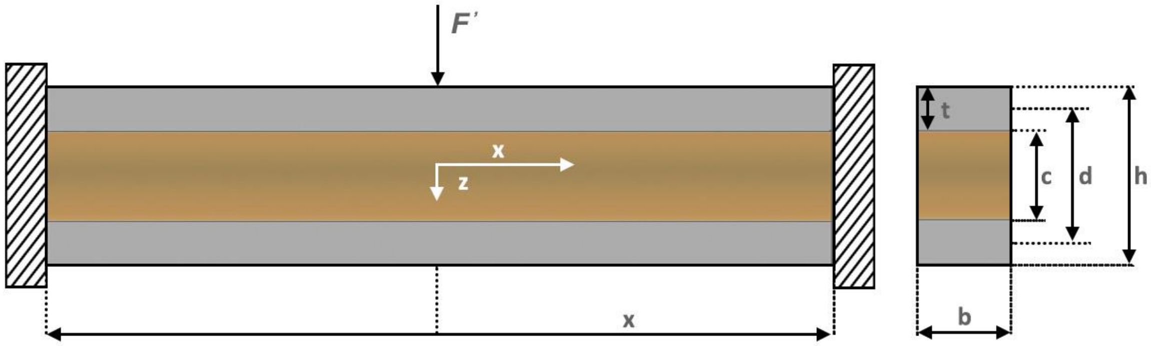

Supplementary Figure 11. Clamped sandwich beam and its side-view.

SUPPLEMENTARY NOTE 2:

Shear strain energy in sandwich structure under centrally pointed load

For simplicity, hypothetical sandwich beam geometries were used to explore the sliding tendencies in 2L graphene and BN. We assume that the beam has a width of a unit cell, length of 1300 nm, and thickness of 0.335 nm for graphene and 0.334 nm for BN, respectively. The geometry of the structure is illustrated in Supplementary Figure 11. The beam is under an imaginary central load (F'). F' was set to the measured fracture load proportional to the fractional area of the sandwich beam used.

Because the interlayer interaction (core) is much weaker than the two faces and the core does not contribute to the flexural rigidity of the structure, shear stress should be constant over the interlayer distance under a centrally pointed load. In other words, the core is considered as an "antiplane" core. The maximal deflection of the beam due to the load is δ. For small displacements, the total deflection of the center of the beam includes the sum of two deflections:[2]

$$\delta(x) = \delta_b(x) + \delta_s(x)$$

where δ_b and δ_s represent pure bending and shear deflections, respectively; x is the distance to the loading center. For a clamped beam under centrally pointed load F':

$$\delta_s(x) = \frac{F'x}{4AG}$$

where G is the shear modulus of the sandwich beam; A is the cross-sectional area of the core. In the isotropic elastic limit, the shear strain energy in the sandwich beam can be calculated by:

$$U_{shear} = \frac{G}{2}\int \gamma^2 \, dV$$

and shear strain (γ):

$$\gamma = \frac{d}{c}\frac{d\delta_s}{dx} = \frac{d}{c}\frac{F'}{4AG}$$

Therefore, we have:

$$U_{shear} = \frac{AG}{2}\left(\frac{d}{c}\frac{F^{'}}{4AG}\right)^2 \int dx = \frac{d^2 F^{'2} x}{32c^3 bG}$$

SUPPLEMENTARY NOTE 3:

Calculation of shear modulus

The shear moduli of graphene and BN are calculated by:[3]

$$G = \frac{d}{\sigma}\frac{\partial^2 U}{\partial x^2}$$

where *G* is shear modulus; *d* is the interlayer distance in graphene and BN; σ is the area per atom in these two materials; *U* is the potential surface of interlayer interaction energy; *x* is distance. σ is estimated using bond length (*l*):

$$\sigma = \frac{3\sqrt{3}l^2}{4}$$

We used the bond lengths of 0.142 nm for G and 0.145 nm for BN, respectively. We can also define:

$$U_{eff} = \left(\frac{l}{2\pi}\right)^2 \frac{\partial^2 U}{\partial x^2}$$

where U_{eff} is the sliding energy in graphene and BN. Therefore, the shear modulus can be calculated by:

$$G = \frac{4\pi^2 d U_{eff}}{l^2 \sigma}$$

It can be seen that G changes when graphene and BN nanosheets are under in-plane strain and out-of-plane compression. In other words, we need to use different values of G to calculate shear strain energy at the loading center and the rest of the suspended nanosheets.

SUPPLEMENTARY REFERENCES

1. Tkatchenko, A. ; Scheffler, M. Accurate Molecular van der Waals Interactions From Ground-State Electron Density and Free-Atom Reference Data. *Phys. Rev. Lett.* **2009**, 102, 073005-073009.

2. Zenkert, D., An Introduction to Sandwich Construction. Engineering Materials Advisory Services: 1995.

3. Lebedev, A. V.; Lebedeva, I. V.; Knizhnik, A. A.; Popov, A. M. Interlayer Interaction and Related Properties of Bilayer Hexagonal Boron Nitride: *ab initio* Study. *RSC Adv.* **2016,** *6*, 6423-6435.
